# Integrating proteomics revealed the key targets for molecular breeding of fine cashmere traits in cashmere goats

**DOI:** 10.3389/fvets.2025.1696953

**Published:** 2025-10-14

**Authors:** Chongyan Zhang, Yichuan Wang, Anru Xing, Bo Xu, Mingkun Wang, Zhixin Wang, Zhihong Liu

**Affiliations:** ^1^Animal Science Department, Inner Mongolia Agricultural University, Hohhot, China; ^2^Inner Mongolia Key Laboratory of Sheep & Goat Genetics Breeding and Reproduction, Hohhot, China; ^3^Key Laboratory of Mutton Sheep & Goat Genetics and Breeding, Ministry of Agriculture and Rural Affairs, Hohhot, China; ^4^Northern Agriculture and Livestock Husbandry Technical Innovation Center, Chinese Academy of Agricultural Sciences, Hohhot, China

**Keywords:** fiber fineness, proteomics, keratin, goat, PRM

## Abstract

In this study, the molecular mechanism of superfine fiber properties of Inner Mongolia Alpas cashmere was revealed by using fiber physical properties measurement and deep proteomics technology. The results showed that the average diameter of Alpas cashmere fiber (14.13 μm) was significantly lower than that of ordinary cashmere (17.29 μm). A total of 79 functional annotation proteins were identified by proteomics analysis, of which 28 were differentially expressed proteins (DEPs). The key findings showed that the down-regulated expression of keratins such as KRT5, KRT14, KRT8, KRT18, and keratin-associated proteins such as KRTAP11-1 and KRTAP15-1 were the core molecular nodes affecting fiber fineness. Functional enrichment analysis showed that these DEPs were significantly concentrated in biological processes such as intermediate fibrous tissue, keratinization, and epithelial cell differentiation, and were localized to keratin filaments. Further studies have found that the enrichment of the estrogen signaling pathway may indirectly affect fiber diameter by regulating the hair follicle cycle, and the interaction between serum albumin (ALB) and keratin group provides a key nutrient transport guarantee for fiber growth. PPI network analysis confirmed that KRT5, KRT14, and other proteins were at the hub of the regulatory network. Parallel reaction monitoring targeted verification ensures the reliability of proteome data. This study proposes that the down-regulation of specific keratins and their associated proteins leads to the formation of finer fibers by finely regulating the differentiation of hair follicle basal cells, cytoskeleton assembly, and keratinization. This study provides an important target for molecular breeding of cashmere goats. By targeting and improving these key genes, it is expected to cultivate new varieties with better cashmere and promote the sustainable development of the industry.

## 1 Introduction

Cashmere trade has a long history, and it was once an important commodity connecting the Cashmere Road of the Eurasian continent and promoting the exchange and integration of Eastern and Western cultures and economies (1). As the world’s largest cashmere producer and exporter, China’s Alpas cashmere goats in Inner Mongolia are famous for producing the world’s top cashmere. The cashmere fiber produced by this variety is soft and uniform, and has won high praise from the global luxury market, and has become a ‘fiber gem ‘in the true sense (2, 3). It not only provides the world’s top brands with the raw materials of dreams, but also wins the international discourse power for China’s cashmere industry, and drives the herdsmen to get rich and increase their income (4).

The excellent properties of cashmere fiber are mainly due to its unique molecular composition. Among them, keratins and keratin-associated proteins (KAPs) are the main structural components of the fiber, accounting for more than 90% of the total. They are assembled to form the skeleton and matrix of the fiber, which directly determines the physical properties of cashmere, such as fineness (3, 5, 6).

In recent years, although genomic and transcriptomic studies have made significant progress in the genetic breeding of cashmere goats, revealing some candidate genes related to fiber traits, as the direct executor of life functions, the relationship between protein expression level and final phenotype is often not completely consistent due to post-transcriptional regulation, translation modification, and other links. Therefore, directly exploring the molecular mechanism of cashmere fiber formation at the proteome level is crucial for the complete analysis of fiber quality formation. However, research in this field is still relatively weak (7).

Therefore, based on the major needs of improving the quality and added value of the cashmere industry in China, this study used high-throughput liquid chromatography-tandem mass spectrometry (LC-MS/MS) technology to conduct in-depth proteomics characterization and comparative analysis of cashmere fibers of different fineness types of Inner Mongolia Alpas cashmere goats. The purpose of this study is to systematically identify the differentially expressed proteins and their interaction networks that play a key role in the formation of cashmere, and to clarify the core proteins and signaling pathways that regulate the fineness of cashmere, to provide new targets for molecular breeding and a theoretical basis for the development of functional cashmere products and ultimately promote the sustainable development of China’s cashmere industry in the direction of high quality and high added value.

## 2 Materials and methods

### 2.1 Animal welfare disclaimer

The Erdos Source Ranch in Erdos, Inner Mongolia, provided grazing conditions for the Inner Mongolian cashmere goats used in this experiment. The Inner Mongolia Agricultural University’s experimental animal management committee has authorized every experimental technique used in this work. The present investigation involved the collection of fiber samples by the International Guiding Principles for Biomedical Research involving animals. The experiment was approved by the Inner Mongolia Agricultural University’s Special Committee on Scientific Research and Academic Ethics, which is in charge of approving the university’s biomedical research ethics [Approval No: (2020)056, project title: the International Guiding Principles for Biomedical Research Involving Animals, approval date: May 6th, 2020]. Two hundred 24 months cashmere goats with similar growth, age, and feeding environment were selected as samples.

### 2.2 Measurement of important economic characteristics

The diameter of the fibers were measured in the laboratory; samples were washed, dried, and tested under conditions of constant temperature and humidity (20 ± 2 °C, 65 ± 4%). Cashmere fiber was cut into short segments, evenly scattered on the slide, on the machine for testing. The diameter was measured using an OFDA 2000 (BSC Electronics, Perth, Australia) optical fiber diameter analyzer according to the IWTO47-2013 standard (Method of Determining Fiber Diameter Distribution Parameters and Percentage of Medullated Fibers in Wool and Other Animal Fibers by the Projection Microscope). EXCEL was used for data processing, SPSS 25.0 was used for one-way analysis of variance and *p* < 0.05 indicated a significant difference.

### 2.3 Protein extraction and digestion

The lysis process involved pulverizing 12 fiber samples in liquid nitrogen (group A, *n* = 6; group B, *n* = 6), adding the powder to lysis buffer (eight milligrams of urea and 1% protease inhibitor), and then lysing the mixture using ultrasound. The supernatant was then transferred to a fresh centrifuge tube after the cell debris was eliminated using a centrifuge set at 12,000 g for 10 min at 4 °C. Following the collection of the filtrate, the processed samples were held at −80 °C while protein quantification was carried out using the BCA ProteinAssayKit (Abcam, China).

### 2.4 Mass spectrometry for label-free liquid chromatography-tandem mass spectrometry

Peptide data were obtained using information-dependent capture (IDA) and sequential window acquisition of all theoretical spectra-mass spectrometry (SWATH-MS) in the Sciex LC-MS/MS system (Framingham, MA, United States). A C18 column measuring 75 μm by 15 cm was used to inject about 2 μg of polypeptide for separation. A linear gradient of 0.1% formic acid in acetonitrile and 0.1% formic acid in water was used to separate the peptides (120 min, 500 nL/min, from 5 to 80%). The IDA parameters were as follows: automated collision energy; 350–1,800 m/z for time-of-flight mass spectrometry collection; 400–1,800 m/z for MS/MS and IDA scan. The nominal resolution was set at 30,000. The following were the SWATH-MS conditions: nominal resolutions of MS1 and MS2, 30,000 and 15,000, respectively; 150–1,200 m/z, MS1 mass range; 100–1,500 m/z, MS2 mass range.

### 2.5 Label-free LC/MS quantitative and qualitative and profiling

Peptide identification was performed using the UniProt/SWISS-PROT/*Capra hircus* database^1^ and Protein Pilot v4.5 software (Sciex, Framingham, MA, United States). A 1% false discovery rate (FDR) was used to filter the results. Trypsin was chosen as the enzyme, and two missed cleavage sites were permitted among the search parameters. There was a 15 ppm peptide mass tolerance and a 20 mmu fragment mass tolerance. The data were imported into the software PeakView v2.1 (Sciex, Framingham, MA, United States), and the SWATH database was searched using the ion library produced by Protein Pilot. PeakView produced the extracted ion chromatograms (XICs) by processing the target and nontarget data. The findings were then explained and subjected to a quantitative analysis using the MarkerView v3.0 program (Sciex). With MarkerView, one may quickly examine data to identify the proteins that are differentially expressed (DEPs). The fold change analysis and t-tests were merged into principal component analysis (PCA) and volcano plot analysis. DEPs were identified using a fold change >2 or fold change <0.5, as well as statistical significance (*p*-value <0.05).

### 2.6 Bioinformatics analysis

Using the *Capra hircus* genome annotation as background and the David database^2^ with default parameters, GO functional and KEGG pathway annotation was carried out. Obtained, encompassing analyses of the cellular component (CC), molecular function (MF), and biological process (BP).

The Search Tool for the Retrieval of Interacting Genes/Proteins (STRING: https://cn.string-db.org/) database was used to perform the protein–protein Interaction (PPI) study of the differentially expressed proteins between goat breeds, as previously mentioned. A minimum needed interaction score of >0.4 was used in the construction of the network. The network was visualized using Cytoscape v.3.9.0 software (Cytoscape Consortium, San Diego, CA, United States). Additionally, the hub proteins of the PPI network were investigated using the Degree approach with the help of the Cytoscape add-on CytoHubba.

### 2.7 Parallel reaction monitoring validation for differentially expressed proteins

The protein abundances were examined using the parallel reaction monitoring (PRM) approach to verify the accuracy of the SWATH-based proteomic data. The peptides were separated using a liquid chromatography-tandem quadrupole mass spectrometry system and dissolved in liquid chromatography mobile phase A (0.1% formic acid solution). The final step was processing the generated MS data with Skyline (64-bit).

## 3 Result

### 3.1 Comparison of the fineness characteristics of cashmere from Alpas goat

**Figure 1:**
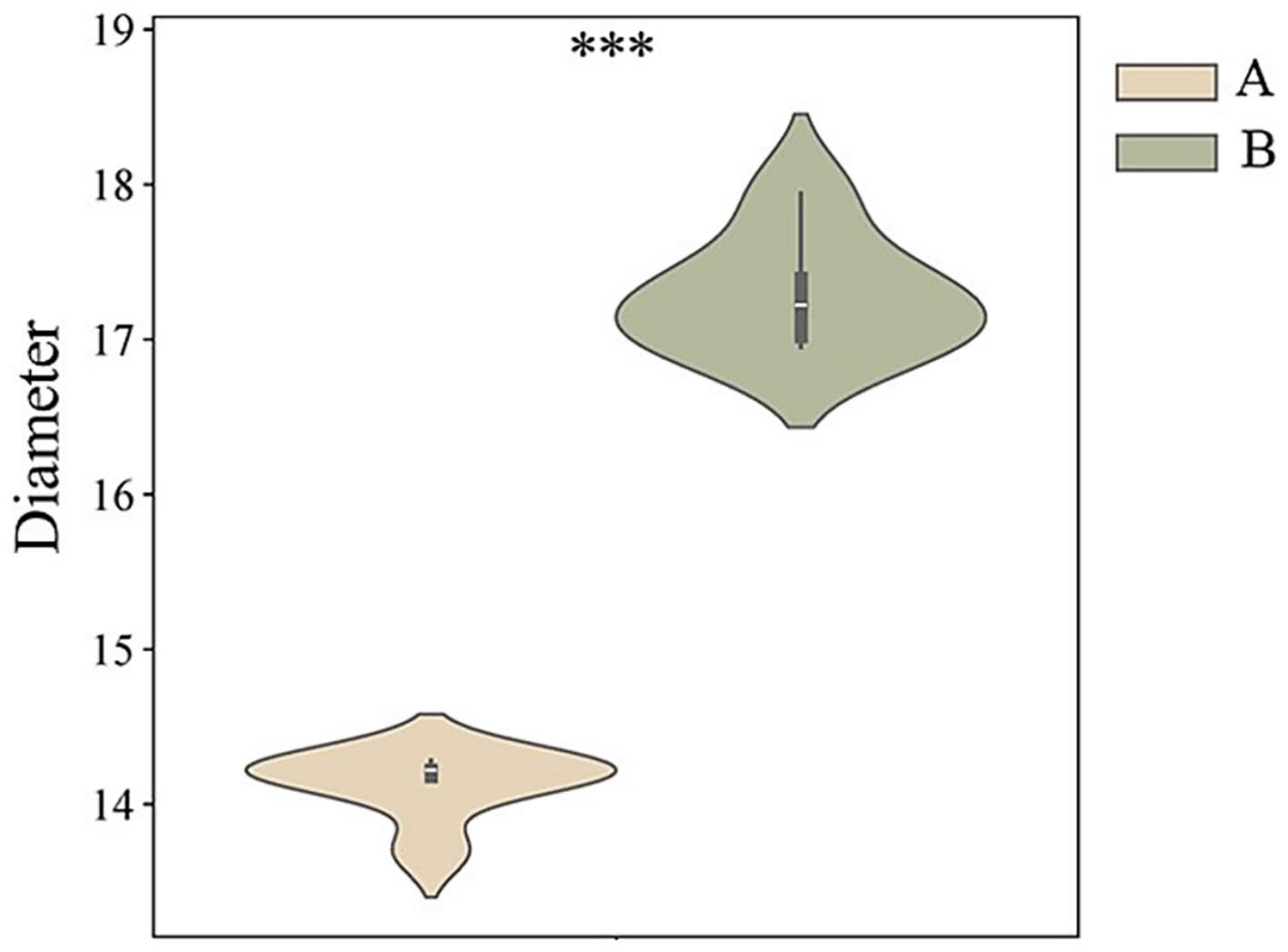
The average fiber fineness of two groups of cashmere fibers.

In this study, the cashmere fiber fineness of two populations (group A and group B, *n* = 100) of Inner Mongolia Alpas cashmere goats was systematically measured and compared. Descriptive statistical results (Table 1) showed that the average diameter of cashmere fibers in group A was 14.13 μm, while the average diameter of cashmere fibers in group B was 17.29 μm. The difference between the two groups was statistically significant (*t* = 1.61 × 10^−6^, *p* < 0.001). The results showed that the Alpas cashmere represented by group A was extremely excellent in the core quality index-fiber fineness, and its fiber was significantly thinner than that of the ordinary control group (see Figure 1).

**Table 1:** Cashmere fiber different fineness data.

Group	A	B
Mean fiber diameter (μm)	14.13	17.29
Fiber diameter standard deviation (μm)	3.70	3.99
Coefficient of variation of fiber diameter (%)	26.18	23.08
Critical fiber diameter (%)	6.92	7.25
Percentage of fiber ≤30 μm	99.90	99.67

### 3.2 Proteomic characterization within the cashmere fiber of goats

**Figure 2:**
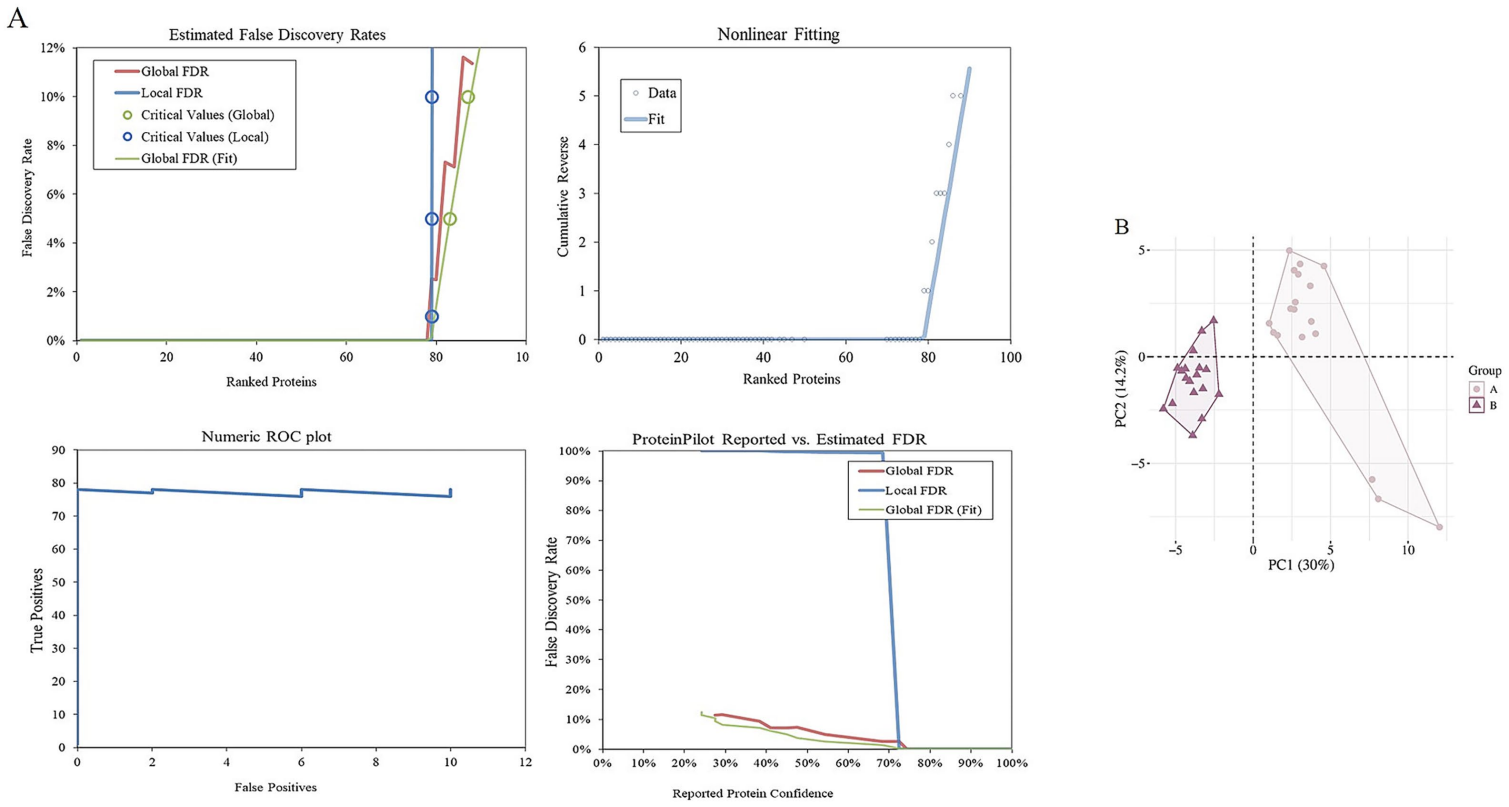
Identification of proteins of fibers from goats. **(A)** Estimated false discovery rates, nonlinear fitting of, numeric ROC plot, and ProteinPilot reported vs. estimated FDR of all proteins. **(B)** Two-dimensional scatter plot of quantitative principal component analysis of protein among samples.

In this study, LC-MS/MS was used to analyze the deep proteomics of two groups of cashmere fiber samples. A total of 4,645 high-quality secondary mass spectra were obtained. After strict data screening and comparison, 4,447 of them were successfully matched to the protein database, and the matching rate of the spectra was 95.7%, indicating that the data quality was high. Based on these high-quality matching results, we identified 847 unique peptide sequences and further systematically annotated 79 proteins. These proteins constitute the potential molecular nodes of fiber diameter variation, which provides an important protein molecular basis for further analysis of the quality regulation mechanism of cashmere fiber development (Figure 2A). In order to evaluate the repeatability and inter-group differences of the whole experiment, principal component analysis (PCA) was performed on the protein expression levels of all 12 samples. The PCA results clearly showed that the samples of group A and group B were clustered in different quadrant spaces, forming a significantly separated cluster distribution, indicating that there were systematic differences in protein expression profiles between the two groups of samples (Figure 2B). On the one hand, it reflects that the experiment has good repeatability and intra-group consistency. At the same time, it is also confirmed from a global perspective that there are essential differences in protein composition between the two groups of cashmere with significant differences in fiber fineness, which provides an important basis for the subsequent search for key functional proteins that cause phenotypic differences.

### 3.3 Functional classification of identified proteins

**Figure 3:**
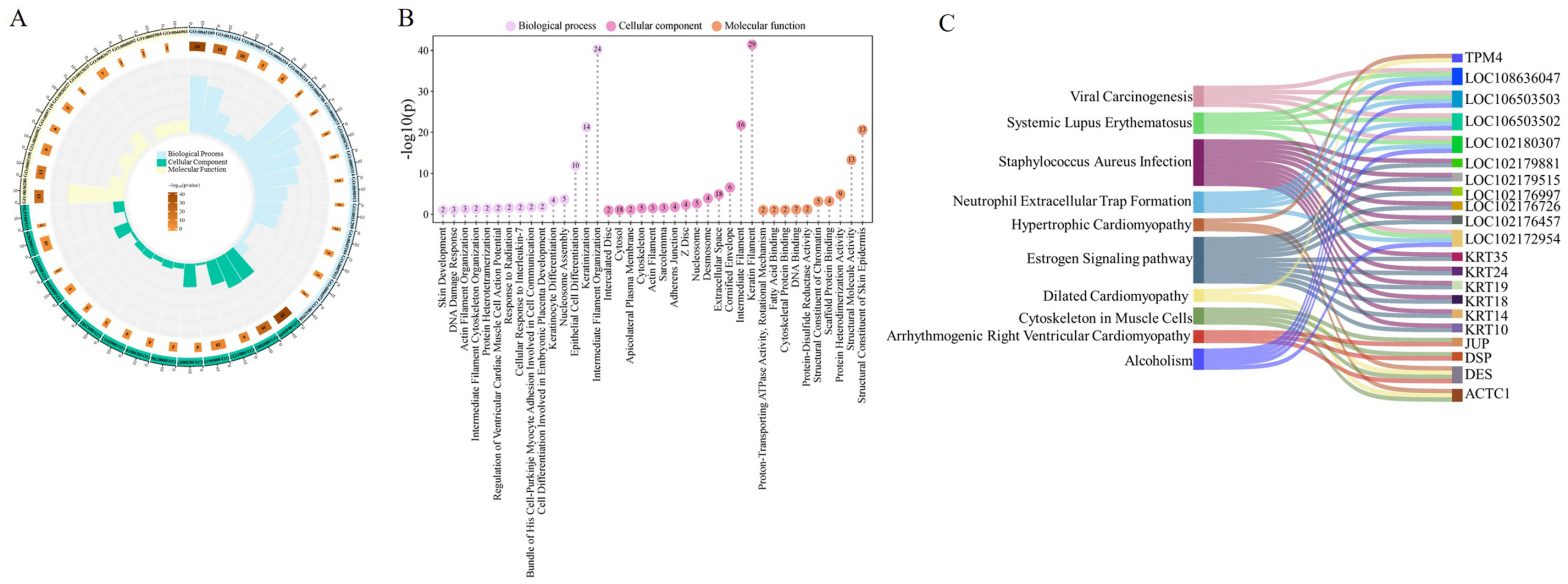
Functional analysis of cashmere fiber protein. **(A)** Cashmere fiber protein enrichment results circle diagram. **(B)** Fibers protein GO function analysis lollipop plot. **(C)** Fiber protein KEGG enrichment pathway mulberry map.

In this study, 79 proteins were systematically annotated by GO function analysis and KEGG pathway analysis databases, aiming to reveal their potential roles in cashmere fiber formation from multiple dimensions such as molecular function, cell localization, and biological processes. A total of 39 significantly enriched functional terms were obtained by GO annotation, covering three main categories: biological process (15), cellular component (14), and molecular function (10) (Figure 3A).

At the level of biological processes, these proteins are significantly enriched in items closely related to fiber structure formation, including intermediate filament organization (GO:0045109), keratinization (GO:0031424), and epithelial cell differentiation (GO:0030855). This result suggests that the protein set screened in this study is mainly involved in and regulates the terminal differentiation and cytoskeleton assembly of fiber keratinocytes, which are the core biological basis for determining the final physical structure (such as diameter) of cashmere fibers. From the cellular component, these proteins are clearly localized in the keratin filament (GO:0045095), extracellular space (GO:0005615), and cytosol (GO:0005829). The localization information further confirms that they act as structural proteins or signal molecules involved in intercellular communication, perform key functions in the interior and microenvironment of the fiber cells, and jointly construct the solid structure of cashmere fibers. Regarding molecular functions, they were found to participate in structural constituent of skin epidermis (GO:0030280), structural molecule activity (GO:0005198), and protein heterodimerization activity (GO:0046982). This indicates that they are not only the physical building blocks of fibers, but also may precisely regulate the assembly mechanism of fibers by forming specific protein complexes (such as keratin heterodimers) (Figure 3B).

In addition, KEGG pathway enrichment analysis revealed that these proteins were involved in multiple potential signaling pathways (Figure 3C). It is worth noting that in addition to the muscle cell cytoskeleton pathway (chx04820) directly related to the cytoskeleton, some pathways related to non-fiber-specific physiological and pathological processes are also significantly enriched, such as the estrogen signaling pathway (chx04915) and *Staphylococcus aureus* infection (chx05150). These pathways may indirectly affect fiber growth and quality by regulating hair follicle cycle, immune microenvironment, or cell stress response. At the same time, the enrichment of multiple cardiomyopathy pathways suggests that some cytoskeletal proteins highly expressed in the heart may also have important structural functions in hair follicle keratinocytes, reflecting the conservation and diversity of cytoskeletal protein functions. The results of this diversified pathway annotation provide new clues and directions for further analysis of the complex regulatory network affecting cashmere fiber growth.

### 3.4 Functional analysis of differential proteins

**Figure 4:**
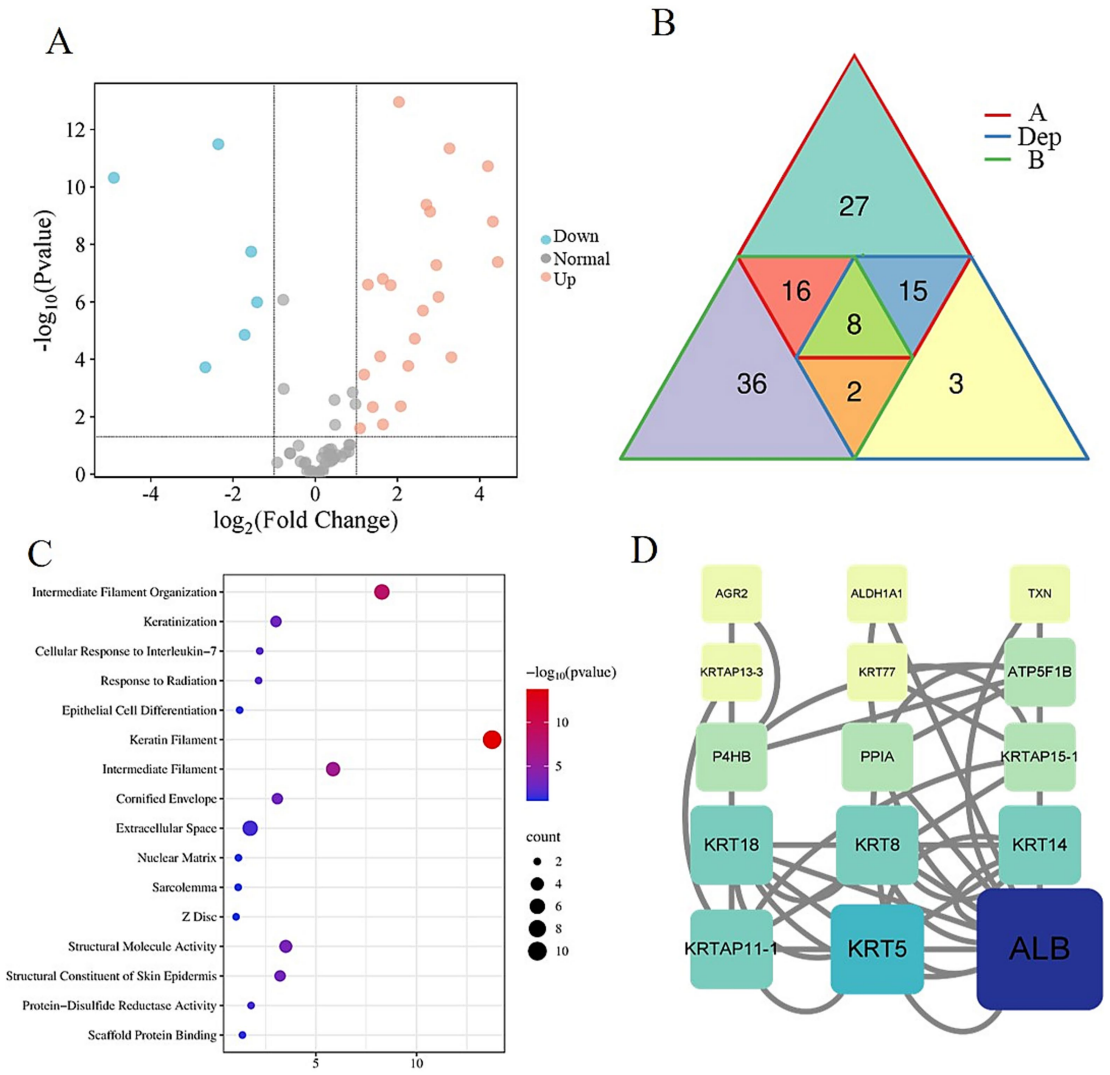
Differentially expressed protein analysis. **(A)** Volcano map of differentially expressed proteins in group A and group B. **(B)** Venn diagram of DEPs, A, and B. **(C)** Bubble plot of differential protein enrichment function. **(D)** Protein–protein interaction regulatory network.

To elucidate the molecular mechanisms underlying cashmere fiber development in Alpas cashmere goats, this study employed LC-MS/MS to construct comprehensive protein expression profiles from fibers of different diameters obtained from 12 individuals. Comparative proteomic analysis between groups A and B identified a total of 28 DEPs, among which 22 were upregulated and 6 were downregulated in the fine-fiber group (Figure 4A). A detailed visualization of the expression patterns and overlap of these DEPs is provided in Figure 4B, highlighting the distinct molecular signatures associated with superior fiber quality. Notably, in group A, six critical proteins exhibited significant downregulation: KRT1, KRT5, KRT14, KRT77, KRT18, and the uncharacterized protein LOC108635630. A similar downregulation trend was observed for five of these proteins (KRT5, KRT14, KRT77, KRT18, and LOC108635630) in group B, suggesting a conserved role in modulating fiber diameter across both groups. To gain functional insights into the DEPs, we performed systematic enrichment analyses. The results demonstrated an association with biological processes and components central to fiber formation. DEPs were significantly enriched in cytoskeleton-related terms, including intermediate filament organization (GO:0045109), keratin filament (GO:0045095), and intermediate filament cytoskeleton (GO:0005882), underscoring their structural roles in fiber development. Additionally, enrichment in the estrogen signaling pathway (chx04915) suggests a potential regulatory mechanism by which hormone signaling may influence fiber growth and characteristics (Figure 4C). Protein–protein interaction (PPI) network analysis further revealed that several keratins (KRT5, KRT8, KRT14, KRT18) and keratin-associated proteins (KRTAP11-1, KRTAP15-1) occupy central hubs within the network (Figure 4D), indicating their pivotal roles in maintaining structural integrity and regulating fiber morphogenesis. Importantly, serum Albumin was identified as a key interactor within this network, suggesting its role as a critical transport molecule that delivers essential nutrients and signaling molecules to support keratin synthesis and fiber growth.

These findings provide novel insights into the proteomic determinants of cashmere fiber quality, highlighting both structural proteins and signaling pathways that contribute to fiber diameter regulation. The integration of differential expression, functional enrichment, and network analyses offers a holistic view of the molecular mechanisms driving cashmere fiber development in Alpas goats.

### 3.5 PRM quantitative results

To confirm the correctness of the proteomic data in three comparison groups, three proteins were chosen for PRM quantification based on the expression level of the DEPs. The findings of the SWATH data analysis and PRM detection ratio showed a steady general trend, suggesting that the proteomic data were dependable and repeatable (Table 2). The results of PRM targeted validation experiments highly reliably confirmed the accuracy of our label-free quantitative proteomics data based on LC-MS/MS. This high repeatability and consistency ensure the solid reliability of all the conclusions based on DEPs in this study.

**Table 2:** PRM result compared with the SWATH-based quantitative result.

Gene name	Peptide	PRM		SWATH	
		A	B	A	B
KRT5	NLQIDPTIQR	374,160	746,250	24.78	48.86
KRT14	EVATNSELVQSGK	342,650	598,450	22.14	39.31
KRTAP15-1	QLGSTLYSDCQENFFRPVSFQTP	302,540	426,540	21.27	32.65

## 4 Discussion

In this study, the molecular mechanism behind the excellent fineness of Inner Mongolia Alpas cashmere was revealed by integrating the physical properties measurement and deep proteomics analysis of cashmere fibers. Our study not only confirmed the significant advantages of Alpas cashmere in fiber fineness but more importantly, revealed the key regulatory proteins and pathways leading to this phenotypic difference from the protein expression level. Keratin is the core structural protein that constitutes the fiber skeleton (8). A series of keratin proteins, such as KRT5, KRT14, KRT8, and KRT18, along with their associated proteins (such as KRTAP11-1 and KRTAP15-1), are key node molecules that affect the fineness of cashmere fibers. Among them, KRT5 and KRT14 form a heterodimer. It is the main structural component of hair follicle basal cells and the outer root sheath, and also a marker of hair follicle stem cells (9, 10). Their down-regulation may imply that the proliferation and differentiation activities of basal layer cells in the hair follicles of group A goats are more refined and coordinated. This could ultimately lead to smaller and more closely arranged cortical cells by influencing the differentiation fate of hair follicle stem cells or the keratinization process of hair matrix cells, thereby forming a finer fiber diameter (11, 12). KRT8 and KRT18 are type I acidic keratin proteins, typically expressed in monolayer epithelium. Their functions in hair follicles have been less studied, but our data suggest that they may be involved in the structural support of the inner sheath of hair follicles or specific cell types. The down-regulation of KRT18 is also associated with a fibrous phenotype. KRTAP11-1 and KRTAP15-1 are high-sulfur keratin-associated proteins that mediate cross-linking between keratin filaments; their down-regulation may reduce filament cross-linking density, leading to thinner fibers. DEPs are significantly enriched in biological processes such as intermediate fibrous tissue, keratinization, and epithelial cell differentiation, and are located on keratin filaments. This strongly proves that the difference in cashmere fiber fineness is essentially determined by the differentiation process of hair follicle keratinocytes and the subtle differences in cytoskeleton assembly procedures. It is particularly noteworthy that the enrichment of estrogen signaling pathways. Estrogen has been widely proven to inhibit the growth of hair follicles and prolong the telogen phase of hair follicles (13, 14). The enrichment of this pathway in group A may imply that there are differences in its signal activity, potentially indirectly affecting the diameter and growth rate of fibers by regulating the cycle and metabolic activity of hair follicles, which provides clues for a new regulatory perspective. PPI network analysis intuitively shows that KRT5, KRT14, KRT8, KRT18, and other molecules are at the central hub of the network. This confirms that these keratins are not only structural components but also the core of the regulatory network. Their expression changes will affect the entire protein interaction network, thereby amplifying its biological effects. In addition, the tight binding of serum albumin (ALB) to keratin groups was detected. ALB is the main transport protein in the blood, which is responsible for transporting nutrients such as fatty acids, hormones, and trace elements (15, 16). Its presence in the fiber and its interaction with keratin indicate that it may serve as an important logistic molecule to directly deliver essential nutrients and bioactive signaling molecules to the growing fiber (17), thereby ensuring the effective synthesis of high-quality cashmere fibers (18, 19).

This study proposes a potential molecular model for finer cashmere fibers in Inner Mongolia Alpas goats: specific keratins (such as KRT5, KRT14, KRT8, KRT18) and their associated proteins (KRTAP11-1, KRTAP15-1) leads to finer and more uniform fiber formation by affecting the differentiation of hair follicle basal cells and stem cells, the assembly of cytoskeleton, and the keratinization process. This process may be regulated by estrogen signaling pathways, and nutritional support is provided by transport molecules such as albumin.

The findings of this study not only deepen our basic understanding of cashmere fiber developmental biology but also provide valuable candidate targets for molecular breeding of cashmere goats. Future research could focus on transcription factors or epigenetic mechanisms that regulate the expression of these key keratin genes. Through gene editing or molecular marker-assisted selection and other technical means, targeted improvement of these sites is expected to cultivate new varieties with higher cashmere yield and better cashmere quality, thus promoting the sustainable development of the cashmere industry.

## 5 Conclusion

This study provides a comprehensive molecular mechanism of proteomics and reveals the key factors of cashmere fineness in Inner Mongolia Alpas goat. By combining the physical properties of the fibers with deep protein analysis, we demonstrated that the special fineness of Alpine cashmere is attributed to the down-regulation of a series of regulatory proteins, including KRT5, KRT14, KRT8, KRT18, KRTAP11-1, and KRTAP15-1. These proteins are the core of cytoskeleton organization, keratinocyte differentiation, and keratin filament assembly processes that fundamentally control fiber diameter. In addition, the enrichment of the estrogen signaling pathway may be a potential regulatory mechanism for regulating hair follicle circulation and fiber growth dynamics, and ALB plays a role in nutritional transport and local signaling in supporting high-quality fiber synthesis.

## Data Availability

The mass spectrometry proteomics data have been deposited to the ProteomeXchange Consortium: https://proteomecentral.proteomexchange.org/cgi/GetDataset?ID=PXD068818 via the iProX partner repository,with the dataset identifier PXD068818.

## Footnotes

^1^ https://www.UniProt.org/#

^2^ https://david.ncifcrf.gov/
